# Addressing quadruple aims through primary care and public health collaboration: ten Canadian case studies

**DOI:** 10.1186/s12889-020-08610-y

**Published:** 2020-04-16

**Authors:** Ruta K. Valaitis, Sabrina T. Wong, Marjorie MacDonald, Ruth Martin-Misener, Linda O’Mara, Donna Meagher-Stewart, Sandy Isaacs, Nancy Murray, Andrea Baumann, Fred Burge, Michael Green, Janusz Kaczorowski, Rachel Savage

**Affiliations:** 1 School of Nursing, McMaster University, 1280 Main Street W., HSC 3N25E, Hamilton, ON L8S4K1 Canada; 2 School of Nursing and Centre for Health Services and Policy Research, University of British Columbia, 2211 Wesbrook Mall, Vancouver, BC V6T 2B5 Canada; 3 School of Nursing, University of Victoria, PO Box 1700, STN CSC, Victoria, BC V8W 2Y2 Canada; 4 Dalhousie University, School of Nursing, Room G26, Forrest Bldg, 5869 University Avenue, PO Box 15000, Halifax, NS B3H 4R2 Canada; 5 McMaster University, School of Nursing, 1280 Main Street W, Hamilton, ON L8S4K1 Canada; 6 Dalhousie University Department of Family Medicine, 8th floor, 8525 Abbie J Lane Building, 5909 Veterans’ Memorial Lane, Halifax, NS B3H 2E2 Canada; 7 Queen’s University Centre for Studies in Primary Care, 220 Bagot Street, P.O. Bag 8888, Kingston, ON K7L 5E9 Canada; 8 Department of Family and Emergency Medicine, University of Montreal, Tour Saint-Antoine, 850, rue St-Denis Montreal, Quebec, H2X 0A9 Canada; 9 Centre de recherche du Centre hospitalier de l’Université de Montréal, Tour Saint-Antoine, 850, rue St-Denis Montreal, Quebec, H2X 0A9 Canada; 10 Dalla Lana School of Public Health, University of Toronto, 155 College St, 6th Floor, Toronto, ON M5T 3M7 Canada

**Keywords:** Canada, Public health, Primary care, Collaboration, Case study, Triple aim, Quadruple aim, Equity

## Abstract

**Background:**

Health systems in Canada and elsewhere are at a crossroads of reform in response to rising economic and societal pressures. The Quadruple Aim advocates for: improving patient experience, reducing cost, advancing population health and improving the provider experience. It is at the forefront of Canadian reform debates aimed to improve a complex and often-fragmented health care system. Concurrently, collaboration between primary care and public health has been the focus of current research, looking for integrated community-based primary health care models that best suit the health needs of communities and address health equity. This study aimed to explore the nature of Canadian primary care - public health collaborations, their aims, motivations, activities, collaboration barriers and enablers, and perceived outcomes.

**Methods:**

Ten case studies were conducted in three provinces (Nova Scotia, Ontario, and British Columbia) to elucidate experiences of primary care and public health collaboration in different settings, contexts, populations and forms. Data sources included a survey using the Partnership Self-Assessment Tool, focus groups, and document analysis. This provided an opportunity to explore how primary care and public health collaboration could serve in transforming community-based primary health care with the potential to address the Quadruple Aims.

**Results:**

Aims of collaborations included: provider capacity building, regional vaccine/immunization management, community-based health promotion programming, and, outreach to increase access to care. Common precipitators were having a shared vision and/or community concern. Barriers and enablers differed among cases. Perceived barriers included ineffective communication processes, inadequate time for collaboration, geographic challenges, lack of resources, and varying organizational goals and mandates. Enablers included clear goals, trusting and inclusive relationships, role clarity, strong leadership, strong coordination and communication, and optimal use of resources. Cases achieved outcomes addressing the Q-Aims such as improving access to services, addressing population health through outreach to at-risk populations, reducing costs through efficiencies, and improving provider experience through capacity building.

**Conclusions:**

Primary care and public health collaborations can strengthen community-based primary health care while addressing the Quadruple Aims with an emphasis on reducing health inequities but requires attention to collaboration barriers and enablers.

## Background

Health systems reforms in Canada are influenced by escalating health care costs, demands of an aging population, increasing prevalence of multiple chronic health and social conditions, and increasing health inequities [1, 2]. Internationally primary care (PC) and public health (PH) collaboration has been touted as a strategy to overcome such challenges [3–6] and is a core feature of the World Health Organization’s vision of primary health care in the twenty-first century (https://www.who.int/docs/default-source/primary-health/vision.pdf). By definition, PC is the first point of entry to a health care system that provides episodic, comprehensive, person-focused care over time, coordinates care by others, and includes health promotion [7]. Although many fee-for-service practices exist, PC in Canada is increasingly delivered by interprofessional PC teams in group practices/networks. Team composition varies and can include physicians, nurse practitioners, registered nurses, pharmacists, occupational therapists, physiotherapists, social workers, respiratory therapists, counsellors and others [8]. PC draws on various blended payment schemes [9] and is generally funded by and accountable to their privately owned practices. A community health centre is a model of primary care delivery in Canada that generally serves vulnerable clients, such as the poor and new immigrants, in geographically defined neighbourhoods. The interdisciplinary team emphasizes population-based and community development approaches to address the social determinants of health of the clients they serve [9] and physicians are salaried.

PH in Canada is defined as, “…fulfilling society’s interest in assuring conditions in which people can be healthy” [10] (p.19). Canadian PH services are generally delivered by multi-disciplinary providers (e.g., public health nurses, nurse practitioners, public health inspectors, health promoters, and epidemiologists) often led by a PH physician. PH is generally funded by and accountable to municipal, regional and/or provincial authorities [11, 12]. Public health functions include population health assessment and surveillance, health promotion, policy development, health protection, disease and injury prevention, and emergency preparedness and response [13, 14].

White argues that an integrated universally accessible health system is built on PC and PH [15]. Primary care and public health collaboration has been the focus of research in Canada to explore integrated community-based primary health care models that best suit the health needs of communities and address health equity [1]. For this study, we used the Public Health Agency of Canada’s definition of collaboration “as a recognized relationship among different sectors or groups, which is formed to take action on an issue in a way that is more effective or sustainable than might be achieved by the public health sector acting alone.” [16] p.9.

Proponents of health system reform have been advancing a framework known as the Triple Aim [17]. The Triple Aim – improving the patient experience, advancing population health, and reducing health costs – is intended to put forth a balanced approach toward improving health for all [18]. Canadian endorsement is widespread, as expressed by the Canadian Foundation for Healthcare Improvement and provincial health ministries [19], regional planning institutions (e.g., Ontario (ON)-based Local Health Integration Networks [20]), and professional associations (e.g., the Canadian Medical Association [21]). Recently, a fourth aim was introduced – improving the provider experience (nurses, doctors and other professionals involved in health care delivery), with providers identified as the backbone of the health system [18, 22]. The Quadruple Aim (Q-Aim) framework has become situated within the forefront of Canadian debates aimed at improving a complex and often fragmented health care system.

Pre-conditions in the health system are being promoted to achieve the Q-aims such as: enrollment of an identified population, commitment to universal health insurance, and a responsible organization or “integrator” [17]. Whittington and colleagues produced a refined list of pre-conditions including: “1) creating the right foundation for population management, 2) managing services at scale for the population, and 3) establishing a learning system to drive and sustain the work over time” [23] (p.265). The integrator determines a collaboration’s purpose, coordinates work, and supports evaluation and learning for capacity building [23]. Further, integrators serve a collaborative function among organizations, particularly under a determinants of health model in which different sectors hold influence.

Wilkinson and colleagues argue that health equity- one of twin moral aims of PH along with population health [24]- should act as the guiding framework to realize the Triple Aim [25] best achieved through collaboration of multiple sectors (e.g., health care, community, and government) to address health and social needs [25]. Martin’s commentary supports this argument:

*In a healthcare world aligned with the Triple Aim, community needs would be continuously assessed and would dictate resource allocation; the social determinants of health would drive a more comprehensive view of the factors contributing to health; and equity across populations would be a driving force in health system reform *[26] (p.59).

The Q-Aim framework was intended to influence macro-level health systems reform. Implementation has often occurred locally where services are delivered, involving multiple organizational partners as demonstrated through International Health Institute’s investigations [23, 27]. Although not initially part of the Q-Aim narrative, PC and PH collaborations present overlapping objectives that can be realized through synergistic partnerships [28], opportunistically placed to achieve Q-Aims [29].

The purpose of this study was to explore the nature of existing PC-PH collaboration in three provinces [Nova Scotia, Ontario and British Columbia], highlighting their aims motivations, activities, barriers and enablers to collaborate, and perceived outcomes. In this paper we discuss how these collaborations can transform the community-based primary health care sector to achieve the Q-Aims.

## Methods

### Case selection and boundaries of the case

A qualitative case study methodology was employed [30]. To ensure diversity, we selected ten cases based on input from our multi-disciplinary, multi-jurisdictional research team and program advisory committee. To be eligible, PH and PC had to work continuously together for at least a year to achieve a service delivery goal. Additional criteria are listed in Table 1. Any other organizations that were involved in the collaboration with PH and PC were also included within the case boundaries. We tested the feasibility of research methods in a pilot case study. Each provincial team conducted three case studies totaling ten case studies.

**Table 1:** Case eligibility criteria

The collaboration must:	
◦ include a PH and a PC organization continually working together to develop and modify strategies to achieve service delivery goals ◦ have begun to act on plans. ◦ have been in existence for at least 1 year since beginning to offer collaborative services ◦ have at least 5 active participants (note: individuals working together in the collaboration with a good knowledge of the collaboration; e.g., managers, practitioners, support staff)	
The above criteria were required for the Partnership Self-Assessment Tool (PSAT) [27] to be valid.	
The collaboration may:	
◦ be working well or not very well ◦ involve multiple organizations, in addition to PC and PH ◦ have provided services in the collaboration on a full or part time basis (e.g., offered twice a week)	

Provincial research leads (RV, SW, MM, LO, DMS, RMM) sent study invitations to PH and PC organizational leads. All agreed to participate. We identified a ‘collaborator’ for each case in each organization to ensure engagement of relevant staff with direct knowledge of the collaboration. There were various types and numbers of providers and managers involved dependent on each case. There was no attempt to ensure representation.

### Multiple data sources and methods

No significant modifications to methods were made following the pilot. We used multiple data sources and methods to ensure methodological rigor. Data collection methods included: the Partnership Self-Assessment Tool (PSAT) [31], focus groups, individual interviews, and document analysis. The research coordinator recruited participants and obtained their written consent to participate in the survey and two focus groups (or interviews if participants could not attend focus groups) and gathered relevant documents. Those familiar with the partnership were recruited including managers, front line, and support staff. The PSAT [31] evaluates partnership synergy, other dimensions of partnerships, perceived benefits and drawbacks, and satisfaction. Criteria for valid results are found in Table 1.

Two, one hour, audio-taped focus groups (focus group A and B) were conducted roughly half a day apart by researchers experienced in qualitative research. Participants were generally the same people for both focus groups. Focus group ‘A’ explored collaboration goals, motivations, activities, processes and structures, and outcomes. For example: What is different about how you deliver services to this population now compared to before this collaboration existed? (See Additional file 1.) PSAT results were shared for focus group ‘B’ and participants reflected on the scores. Questions included: How does this (PSAT) score resonate with what you perceive about this collaboration? Why do you think your collaboration received this score? (see Additional file 2.) Refreshments and a gift card were provided to participants. We collected relevant collaboration documents (e.g., minutes, logic models, evaluations) for analysis.

### Analysis

Experienced qualitative researchers (conducted multiple qualitative studies) with backgrounds in PC, PH or both analysed the transcribed focus group recordings and text-based data using a descriptive qualitative approach supported by NVivo10. Data were coded inductively, loosely organized by study aims. This was followed by reorganizing codes into higher level categories (e.g., precipitators and activities). The research team met to work through some transcripts to establish the coding structure. Each province was then responsible to code their cases within this structure. The team met multiple times via web conferencing to merge coding and edit the coding structure as needed. NVivo matrix queries were conducted to highlight commonalities and differences across cases.

Trustworthiness [32] was enhanced through multiple data collection methods and data sources (dependability); thick descriptions of cases (transferability); peer debriefing among the research team and the use of illustrative quotations (credibility); an audit trail of decisions (i.e., captured through memos); and triangulation of results from focus groups, interviews and documents (confirmability).

## Results

### Participants

Table 2 provides information about participants and data sources by case. In one case, focus groups ‘A’ and ‘B’ were combined per the organization’s request. Forty-two focus groups and 12 individual interviews were completed. Seventy-three people participated in focus group A, 80 in B, and 8 in the combined focus group. Overall, there were 328 participants including 59 physicians, 182 nurses, 8 occupational therapists, 12 business administrators, and 67 others (e.g., nurse practitioners, community developers, social workers, occupational therapists). PSATs were completed by 7 to 14 participants per case (total *n* = 98) with a completion rate ranging from 36 to 100% per case. Seven cases had a response rate of 65% or greater within 30 days required for valid results [31].

**Table 2:** Number of participants by sources of data and case

Data collection	Case 1: Enhanced 18 Month Well Baby	Case 2: Comprehensive Tobacco Cessation	Case 3: Regional E-Health for Immunization Management	Case 4: Vaccine Management & Information Exchange	Case 5: Rural Community Health Initiative	Case 6: Women’s Health Promotion	Case 7: Rural Youth Health Promotion	Case 8: Urban Child Health Promotion & Family Outreach	Case 9: Inner City Outreach	Case10: Street Health Outreach
**PSAT respondents (n; %)**	(10; 67)	(8; 67)	(8; 89)	(8; 53)	(9; 64)	(14; 82)	(11; 100)	(11; 79)	(12; 36)	(7; 78)
**# of Focus groups “A’; # Interviews**	2; 0	3; 0	2; 0	2; 0	1; 1	1; 3	4; 0	2; 2^b^	1^a^	2; 2
# Participants										
◦ `Front line staff	*n* = 6	*n* = 5	*n* = 3	*n* = 2	*n* = 6	*n* = 2	*n* = 10	*n* = 7	Total *n* = 11	*n* = 3
◦ Managers/physician leads	*n* = 9	*n* = 2	*n* = 3	*n* = 2	*n* = 2	*n* = 3	*n* = 1	*n* = 1		*n* = 3
**# of Focus groups “B′; # Interviews**	4; 0	2; 0	2; 0	2; 1	1; 1	3; 1	4; 0	^b^	2 ^a^	2; 1
# Participants										
◦ Front line staff	*n* = 5	*n* = 3	*n* = 3	*n* = 5	*n* = 7	*n* = 6	*n* = 10	See Focus group A	Total *n* = 15	*n* = 2
◦ Managers/physicians	*n* = 8	*n* = 2	*n* = 3	*n* = 3	*n* = 1	*n* = 3	*n* = 1			*n* = 3
**# of Documents**	8	20	20	14	5	20	5	4	2	3

^a^ front line and managers combined in a focus group | ^b^focus groups A and B were combined

### Overall results

Professional disciplines involved in collaborations included: public health nurses (PHNs), nurse practitioners (NPs), family practice nurses, PC physicians, Medical Officers of Health (i.e., public health physicians), mental health workers, administrators and managers, occupational therapists (OT), speech therapists, social workers, midwives, information technology experts and operations staff (e.g., clerks, receptionists). Populations served often had limited access to PC or PH services and/or were disadvantaged due to lack of stable housing, poverty, discrimination and stigmatization, poor mental health, trauma, or violence. Collaborations also engaged in immunization programs and vaccine management that served the population-at-large. Other populations included youth, women, or adults in rural communities living with mental health problems or addictions. Some cases focused on building service provider capacity in health promotion.

Collaborations, at times, developed organically in response to community needs and a mutual sense of responsibility to address them. In other cases, they developed formally as partnering agencies worked together on strategic plans, set goals, identified priorities, and participated in steering committees. In some cases, goals and priorities were refined over time through ongoing meetings with providers and community groups.

There were varying precipitators that supported initiating a collaboration. Potential partners often shared a common vision and/or community concern. They perceived that working together could have a greater impact by using resources differently, addressing community problems together, or offering alternative solutions to meet service demands. Some collaborators saw opportunities to increase effectiveness and/or maximize efficiencies, since they worked with the same populations. Tipping points that enabled action on collaboration related to provincial funding incentives for new initiatives or ways of practicing to address common concerns.

There were four key foci for collaborations. These included: provider capacity building; regional vaccine /immunization management; community-based health promotion programming; and, increasing access to care through outreach programs and services. We identified common inter and intrapersonal, organizational and systemic enablers and barriers to collaboration. Common enablers included personal skills, knowledge and attitudes that supported the collaboration, clear roles, effective communication and coordination strategies, strong organizational leadership, formal agreements, human resources, and provincial mandates that were aligned with collaboration aims. Common barriers included turnover of staff, lack of role clarity, lack of resources and funding for collaboration, and a lack of support from the provincial government.

### Detailed case descriptions

Detailed results are presented by case (see Additional file 3) describing aims and motivators for collaboration, provider activities, and perceived outcomes. In addition, we present key collaboration barriers and enablers organized under systemic, organizational, interpersonal and intrapersonal factors influencing collaboration. Cases were organized by categories based on common goals including: Cases 1 to 2 - provider capacity building; Cases 3 to 4 - regional vaccine /immunization management; Cases 5 to 7 - community-based health promotion programming; and, Cases 8 to10 - increasing access to care through outreach programs and services. Relationships to the Q-Aim framework are highlighted. Table 3 illustrates the intensity of activities (i.e., minor focus [+], moderate focus [++], or major focus [+++]) conducted in each case demonstrating *how* they worked together. A description of each case by category follows and the most compelling links to the Q-Aim framework are acknowledged. Sources of quotes are identified by case number, sector (PH, PC, Community or both PC and PH), and discipline (e.g., PHN, MD, Other).

**Table 3:** Activities of Primary Care and Public Health Collaborations by Category and Case

+ Minor focus ++ Moderate focus +++ Major focus										
Categories	Provider Capacity Building		Regional Vaccine /Immunization Management		Community-based Health Promotion Programming			Increasing Access to Care through Outreach Programs & Services		
Collaboration Activities	Case 1: Enhanced 18 Month Well Baby	Case 2: Comprehensive Tobacco Cessation	Case 3: Regional E-Health for Immunization Management	Case 4: Vaccine Management & Information Exchange	Case 5: Rural Community Health Initiative	Case 6: Women’s Health Promotion	Case 7: Rural Youth Health Promotion	Case 8: Urban Child Health Promotion & Family Outreach	Case 9: Inner City Outreach	Case10: Street Health Outreach
Community betterment/ engagement		+			+++	++	++	+++		
Provider capacity building	+++	+++	++	++	+		++	+	++	+
Enabling access to care/services	++	++	+++		++	+++	+++	+++	+++	+++
Health promotion	++	+			+++	++	+	++	++	
Prevention	+++		+++		+			+	+++	++
Protection							+		+++	+++
Harm Reduction		+++							+++	+
Health Education	+++	+++			++		+	++	++	
Surveillance	++		+++	+++			+	+++	++	
Joint Program and Service delivery			+++	+++	+	+	++	++	+++	+
Outreach						++		++	+++	+++
Sharing of Information Resources	+++	++	+++	+++	+++	++	+	+++	++	++
Acute/ Episodic care								++	+++	
Chronic Disease management	+				+				++	

### Provider capacity building

In two cases, partners engaged in provider capacity building to address client needs. Improving provider confidence through professional training can indirectly address the Q-Aim – improving the provider experience and ultimately patient experience.

Case 1 began with PC’s desire to develop provider capacities in working with at-risk children. A PHN was seconded to the PC practice located in a large urban setting with multiple practice sites to build PC provider capacity in conducting enhanced 18-month well baby assessments. The collaboration also was a means of rekindling historically positive PC and PH relationships that could lead to future collaboration:

*… Both [PH and PC] felt that the relationship between the two sectors had eroded and historically we’d had a very close relationship. […] So whatever strategies we were using to date weren’t working. […] So, the collaboration was really designed to address all of those things. [Case 1 PH-Other]*

Enablers were related to high levels of trust between PC nurses and the PHN, having a formal contract, and funding for the secondment. Barriers included physicians’ perceptions of being excluded from the development of the collaboration and the lack of compensation and protected time for PC nurses to attend training.

Case 2 involved a PHN working closely with a PC NP in a rural PC practice with satellite locations to train PC staff on comprehensive tobacco cessation. Provincial funding for staff and resources provided the tipping point for the collaboration. Mutual interests determined collaboration goals.

*… “our combined goal was really to get a good comprehensive strategy embedded in the [PC team] and to reach out to the community as well… As [NP] was saying, to educate front line staff so that everybody was coming from the same place and had a good solid understanding.*” [*Case 2 PH-PHN*]

Collaboration enablers included: a clear provincial mandate for both sectors to work on tobacco cessation, clear roles, strong past relationships, shared material resources and space, as well as a local award celebrating the collaboration. As in Case 1, there were inequities for PC nurses who were paid through a special funding envelope that made them ineligible to attend training.

### Regional vaccine and immunization management

Two cases focused on increasing immunization coverage rates for geographically distinct regions. They addressed two Q-Aims - improving population health and reducing costs by creating operational efficiencies.

Case 3 involved coordination of a regional flu campaign using a shared electronic health record and appointment system. A PC organization serving most residents in a small northern community collaborated with PH to enhance immunization coverage supported by community members. Participants saw collaboration as a means of reducing duplication while improving efficiencies and addressing partner’s reporting needs. Previous ineffective immunization campaigns highlighted the need to collaborate:

*…the collaboration started out of desperation, to be honest. We needed to get vaccine into people’s arms and neither one of us could meet the demand... […] I’m sure it came out of discussions at meetings… [Case 3 PH-PHN]*

Enablers included strong relationships and trust among providers and community members who had previous working relationships, a common vision among organizational leadership, a formal partner agreement, provider training on the Electronic Medical Record system to track immunizations, optimizing human resources (e.g., PC expertise in IT systems and PH’s expertise in immunization). Barriers included the legacy IT system and a lack of community volunteer engagement to assist in implementing the campaign.

Case 4 served a mixed urban-rural region and involved PC and PH exchanging paper-based immunization records to increase accuracy in records, tracking immunization coverage, and reducing vaccine wastage.

*…we have the hope that within x number of years, we will have immunization information on every child who’s in school. That automatically will come to us because of the partnerships that we have now. [Case 4 - PH-PHN]*

A PH driver delivered vaccines to participating PC practices, exchanged immunization records, inspected for cold chain breaks, and connected PC staff to PHNs in the communicable disease program to answer clinical questions. A manager reported significant reductions in vaccine wastage and recognized opportunities to improve information systems through cooperation.

Enablers included positive personal characteristics of the PH and PC providers (e.g., knowledgeable), effective interpersonal communication, strong coordination and communication processes, PHNs assigned to work with PC, strong PH leadership that included conflict management skills. A barrier was the time required to build PC PH relationships in the region.

### Community-based health promotion programming

Three cases involved community-based health promotion programming in rural communities that addressed two Q-Aims – improving the patient (client) experience and improving population health. Case 5 involved a solo practice physician working with community agencies (i.e., PH, community members, researchers, local and regional governments, NGOs, First Nations communities, and parks and recreation) in a geographically-dispersed rural setting. The collaboration focused on youth health, mental health, food security and social determinants of health. A steering committee consisting of community members and service providers was instrumental in spearheading the collaboration. Terms of Reference included access and inclusiveness goals and collaborators promoted a seamless network to improve care processes:

*… to make it easier for all the information to get around to the various organizations and for them to collaborate or for them to network*. *[Case 5 Community-Other]*

Recognition of community needs and service gaps and research funding helped solidify working relationships to address population health:

*... a group of community service providers got together to address the abysmal lack of mental health and addictions services in the community. From that table … arose the idea of a community-based participatory action research project. So, over a course of two years we acquired funding and developed a new role and a community mental health service access and a mental health service navigation… [Case 5 PC-MD]*

Collaboration enablers included individual skill sets and personal commitment to address the common goal, appreciation shown for volunteers, open and transparent discussions, and collaboration champions. Barriers included varying organizational goals and philosophies, the informal collaboration structure leaving it vulnerable, challenges in scheduling geographically dispersed meetings, and competition for scarce system level resources.

Cases 6 and 7 aimed to improve access to health promotion and illness prevention for specific populations through a client-centred approach by matching resources to individual, family, and community needs. Case 6 focused on rural women, and like Case 5, motivators included concern for families ‘falling through the cracks’, inequities, and gaps in services:

*It was an opportunity to provide services to women in a better way, in a more responsive way to [address] the needs of the individual in a setting that was more comfortable for the individual person coming in. […] So, it was really around trying to do a better job for an under-served population of women and adolescent girls*. *[Case 5 PC-Other]*

A non-government organization led the collaboration among PC, PH and other agencies in a shared space. Enablers included individuals’ strong belief in and commitment to women-centred care, flexible roles that matched providers’ skills, having formalized agreements and operational plans for the collaboration, and regular committee meetings. Barriers included finding a good fit for PH providers in a PC setting, high staff turnover, a lack of cash resources, and system level mandate changes resulting in provider role confusion.

Case 7 emphasized infant, child, and youth health. Collaborators conducted joint planning to address immunization program inefficiencies while offering comprehensive programming working with community members. This rural collaboration developed through formalized conversations that helped partners recognize common community concerns:

*It’s that groundwork that you need to do upfront, setting those goals, coming as a collective, having those conversations that bring you to the same place, having a common commitment and understanding […]of what needs to happen. [Case 7 PH-Other]*

Enablers were joint training and meetings, and leadership to drive the collaboration. Barriers included conflicting PC and PH mandates and changes in mandates that contributed to role confusion. Similar to Case 6 the collaboration had high staff turnover and PC struggled to find time for the collaboration due to heavy work demands.

### Increasing access to care through outreach

Case 8, 9 and 10 applied an equity lens to increase access to services for hard-to-reach populations through outreach best aligning with the Q-Aims – improving the patient experience and population health. Case 8, a social pediatrics initiative provided outreach services emphasising health promotion for at-risk children and families (i.e., poor, exposed to substance use and/or family violence) in a large urban centre. Service gaps for hard to reach, at-risk families, and a lack of PC physician access motivated PC NPs to provide outreach and service coordination. They offered services to young families at community locations (i.e., schools), and referred to a tertiary care centre for specialist services, PH and other health and social services:

*We’re trying to make it a low barrier system so that if you go into the community center and you need health care, the community center can help you get to health care. If you go into PH and get immunizations and you need some developmental assessment or you need some kind of maternal mental health assessment, you will get linked that way. […] every door is a way in. [Case 8 Community-OT]*

Enablers included individuals’ personal commitment to the initiative, a publicly shared role definition for the NPs, knowledge of who to approach to address issues, and engagement of partners and community members/clients at community tables. Barriers included differing philosophies and communication modes among partners, a lack of leadership buy-in, and no overall leader.

Case 9 was an inner-city outreach program for street-involved population. A coalition of community organizations, including PC and PH, followed a project charter with service objectives including communicable disease control, outreach, disease prevention, treatment and referral, addictions and mental health counselling. Shared concerns for those without access to services moved PC and PH providers to collaborate without formalized relationship agreements:

*...we haven’t embedded routine sharing arrangements; that sort of happened almost automatically. [Case 9 PH-MD]*

Enablers included capable, skilled front-line staff, respectful interpersonal relationships, and interdisciplinary teams that brought different strengths. Barriers included a lack of role clarity between PC and PH providers exacerbated by changing PHN roles, and no common communication infrastructure.

Another urban outreach program, Case 10, served a street-involved population focused on improving immunization coverage against influenza. PHNs gained access to ‘the street’ through PC nurses who were trusted in the community. PC nurses, PC and PH physicians, PHNs, managers, and administrators shared a passion for equity and social justice and strong desire to reach the underserved:

*… this is the population that nobody else really takes care of. And so [managers and directors] are very supportive of us providing these services and involving our front-line practitioners, or our PHNs to be involved. [Case 10 PH-Other]*

Enablers included individuals’ passion and skills in working with marginalized communities and a commitment to equity and social justice. Previous working relationships were helpful, and a lack of a formal agreement allowed for more flexibility in the collaboration. Communication was informal challenging busy workloads. In-kind resources were enablers given the lack of provincial level funding for the collaboration.

### Impacts and outcomes of collaborations

Published papers reported on outcomes for two cases (not cited to protect confidentiality). Other cases included plans for evaluation supported by an evaluation framework or a logic model. Participants shared a range of perceived impacts and outcomes. Most cases appeared to have achieved multiple outcomes relating to Q-Aims.

Improved outbreak management (Cases 1, 3, 9, 10) and enhanced harm reduction (Case 2) were perceived to have achieved safer care for the population, a condition required for a quality health services system [33] as well as improvements in population health. Quality of services was increased as clients benefited from services offered through PC-PH collaborations (Cases 1, 2, 3, 6). For example:

*Definitely PH’s involvement in the 18-month visit did change the way that we deal with the 18-month visits and in general- baby visits. We’ve changed and have become a little bit more creative, more open to new things and whatever can help us do things better and help the parents with their child better. [Case 1 PC-RN].*

Improved service delivery models that included program expansion were achieved through shared services, access to information technology, and record keeping redesign (Cases 3, 4 and 10).

*The health centre has been doing immunizations for the marginalized through our nursing services for quite some time… What [street outreach] does though is allow for expansion of that. [Case 10 PC-MD]*

Participants perceived that there were service improvements related to continuity, reliability and responsiveness. Work processes were enhanced through the development of support networks that enabled access to resources and enhanced communication among partners, thereby improving patient experience:

*…whenever we have a new case who is infectious, we case conference and include all of the relevant players […] so that we’re all on the same page. [Case 9 PH-PHN]*

Timeliness of services was enhanced through reduced wait times (Case 4) and increased person-centred care (Case 6 and 7). Participants reported improved relationships between clients and providers (Case 9).

From a population health perspective, 8 of 10 cases reported improved access to services for marginalized populations to address health inequities (Cases 1, 2, and 5 to 10). This was achieved through inter-agency referral and communication, joint programming to improve service efficiencies, and advancing outreach activities:

*But that group of marginalized people would not have been immunized if [street outreach] and PH did not have that relationship. They would have had to wait to get into their family doctor. And they wouldn’t have gotten in because they wouldn’t have necessarily had a family doctor. [Case 10 PH-Other]*

Participants in Cases 5 and 8 reported policy impacts at a municipal/regional level.

*It did affect public policy in terms of getting [the district] to look at the development of walking trails... [Case 5 PH-PHN].*

Other collaborations paved the way for policy change.

*And that’s why we were able to champion the H1N1 flu vaccine for that population specifically and just do it. Which is basically changing public policy because we just did it, and it was against what we were being told to do at the provincial level. So, I think some of that public policy stuff, this partnership has enhanced it. [Case 10 PH-Other]*

Other population health impacts included: increased immunization rates and enhanced ability to respond to epidemics (Cases 7 and 10), enhanced awareness of community health problems (Cases 6 and 9), reduced tobacco use (Case 2), and a shift to a population focus (Cases 7 and 9).

A few cases reported perceived cost-reducing efficiencies through shared programming, record keeping, or delivery of vaccines to PC offices based on use (Cases 3, 4). Costs savings were realized through better resource allocation, reduced vaccine wastage, resource sharing (e.g., IT systems), and in some cases, reduced workloads by avoiding duplication:

*So, we decided that, why can’t we have Public Health go with the family practice nurses and nurse practitioners and run a clinic in the primary healthcare clinics across [the county]? So, we organized that last year. […]. It had bumps but for the most part, it reduced the need for additional resources. [Case 3 Both-Other].*

In relation to the provider experience, staff knowledge and skills increased including a stronger understanding of partner roles and functions, valuing of roles, and improvements in evidence-informed practice:

*PH has recognized our connection and our relationship to that community […] ‘Okay, how do we work with you since you’re going to do this?’ What can we do to make your job easier but also to increase the components around prevention, treatment and care in the community? [Case 10 PC-RN]*

There were positive impacts as a result of relationship building, such as improved accountability:

*… When you meet on a front-line level, I think there's a different accountability.... […] there is an accountability piece that happens there because we've entered into this relationship and we've agreed that we will service these women in a very distinct way…* [Case 6 PC/PH-Other]

There also were positive spin offs from other agencies’ contributions to collaborations:

*So, the NP and the physician, they were forced to deal with a lot of non-medical PC issues to get people there. So, I think their scope really expanded because they could work with the [NGO] where advocacy is one of their main roles and learn from them. [Case 6 PC/PH-Other].*

### Drawbacks and benefits of collaboration

Multiple benefits from collaboration were perceived as it relates to patient experience:

*It’s quite a powerful synergy when you have PC and PH rubbing shoulders together. … if there’s the interchange of ideas, there’s also a much better experience for the client to be able to access all of those things […] in a much more powerful way. [Case 9 PC/PH-MD]*

In terms of provider experience job satisfaction was improved in some situations despite a lack of compensation for added responsibilities:

*… financially [collaboration is] a disadvantage […]. I think we’d do it anyways just for our own personal satisfaction. [Case 1 PC-RN]*

Participants reported few drawbacks. A few perceived collaborations to benefit some partners more than others, particularly if collaborations appeared to divert resources away from valued services or if collaborations added to busy PC or PH workloads (i.e., provider experience).

**Fig. 1:**
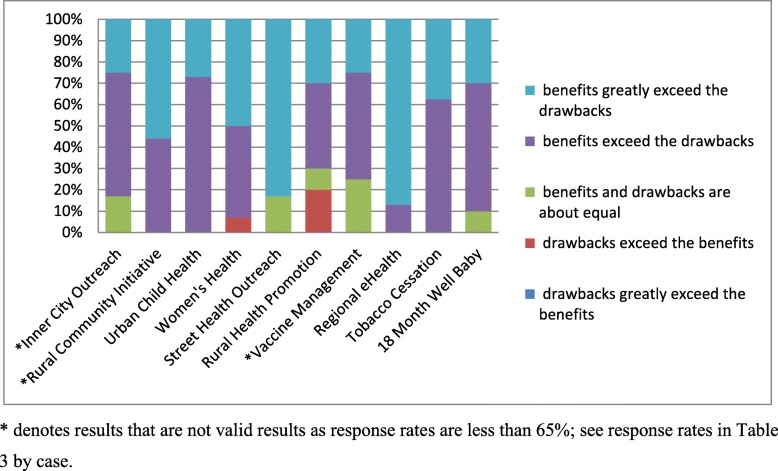
Benefits versus drawbacks of participation

In all cases, the majority of PSAT respondents reported that collaboration “benefits exceeded the drawbacks” or “greatly exceeded the drawbacks” (Fig. 1). The majority attributed benefits of collaboration to (Table 4): the development of valuable relationships (provider experience), enhanced ability to meet the needs of my constituency or clients (patient experience), ability to make a greater impact than I could have on my own (patient/provider experience), ability to make a contribution to the community (population health/patient experience), and enhanced ability to address important issues (population health/provider experience). Two items that helped explain drawbacks were: time diverted from other activities (patient/provider experience), and frustration or aggravation (provider experience) (Table 5).

**Table 4:** Benefits of partnership (Percentage Rating Agreement by Case)

	Provider Capacity Building		Regional Vaccine Immunization Management		Community-based Health Promotion Programming			Increasing access to care through outreach programs and services		
Benefits Response Rate (RR)	Case1 *n* = 10 (RR = 66.7%)	Case2 *n* = 8 (RR = 66.7%)	Case 3 *n* = 8 (RR = 88.9%)	Case 4 *n* = 10 (RR = 53.3%)^a^	Case 5 *n* = 9 (RR = 64.2%)^a^	Case 6 *n* = 14 (RR = 82.4%)	Case 7 *n* = 11 (RR = 100%)	Case 8 *n* = 11 (RR = 78.6%)	Case 9 *n* = 12 (RR = 36.4%)^a^	Case 10 *n* = 7 (RR = 77.8%)
Enhanced ability to address important issues	89	88	88	75	78	86	82	82	92	100
Development of new skills	89	75	88	50	78	71	55	55	92	86
Heightened public profile	44	100	100	50	67	86	18	64	75	83
Increased utilization of my expertise or services	70	63	100	50	89	86	73	73	83	83
Acquisition of useful knowledge about services, programs, or people in community	80	100	100	75	100	77	45	82	92	100
Enhanced ability to affect public policy	33	38	63	13	44	36	9	36	58	33
Development of valuable relationships	90	100	100	75	100	100	91	100	92	100
Enhanced ability to meet the needs of my constituency or clients	80	88	88	75	78	93	82	91	100	100
Ability to make a greater impact than I could have on my own	90	100	100	88	89	93	82	100	92	100
Ability to make a contribution to the community	100	75	88	88	100	93	73	91	75	100
Acquisition of additional financial support	22	63	38	0	44	29	18	27	33	0

^a^ invalid results – response rate (n/N) less than 65%

**Table 5:** Drawbacks of partnership (Percentage Rating Agreement by Case)

	Provider Capacity Building		Regional Vaccine Immunization Management		Community-based Health Promotion Programming			Increasing access to care through outreach programs and services		
Percentage	%	%	%	%	%	%	%	%	%	%
Drawbacks Response Rate (RR)	Case 1 *n* = 10 (RR = 66.7%)	Case2 *n* = 8 (RR = 66.7%)	Case 3 *n* = 8 (RR = 88.9%)	Case 4 *n* = 10 (RR = 53.3%)^a^	Case 5 *n* = 9 (RR = 64.2%)^a^	Case 6 *n* = 14 (RR = 82.4%)	Case 7 *n* = 11 (RR = 100%)	Case 8 *n* = 11 (RR = 78.6%)	Case 9 *n* = 12 (RR = 36.4%)^a^	Case 10 *n* = 7 (RR = 77.8%)
Diversion of time and resources away from other priorities or obligations	60	50	63	38	67	64	55	0	67	27
Insufficient influence in partnership activities	38	0	25	25	11	14	64	0	50	45
Viewed negatively due to association with other partners or the partnership	0	0	25	14	0	14	9	0	8	9
Frustration or aggravation	50	13	50	38	33	64	40	17	58	45
Insufficient credit given to me for contributing to the accomplishments of the partnership	10	0	25	29	0	7	36	0	25	0
Conflict between my job and the partnership’s work	40	13	38	13	33	21	18	0	33	0

^a^ invalid results – response rate (n/N) less than 65%

PSAT satisfaction (provider experience) responses indicated that participants, other than rural health promotion (Case 7), were generally satisfied (completely or mostly) with: a) working together (range 33–100% of participants per case; average 74.5%), b) role (range 59–88%; average 75%), c) influence (range 50–100%; average 74%), and d) plans (range 50–100%; average 74%). In Case 7, general satisfaction scores ranged from 9 to 40 and 20% of participants indicated that drawbacks exceeded benefits. These results may explain some of the high staff turnover in this collaboration.

## Discussion

The case studies demonstrate that collectively PC and PH collaboration can help to address Q-Aims. Our research provides evidence that collaboration between PC and PH can work to address: specific health issues (immunization), outreach to increase access to services for vulnerable populations, community-based health promotion and prevention programming for specific population groups (e.g., women, youth), and provider capacity building. Participants reported PC and PH collaborations worked to: improve access to services for patients through joint programming and information sharing, address population health through outreach to at-risk populations under a social justice and equity framework, reduce costs through efficiencies, and improve the experience of providers through shared learning and capacity building and mutual respect/recognition. For the majority, benefits of collaboration outweighed drawbacks. Despite reported concerns about draining PH resources to PC in collaborations [1, 34], diversion of public health resources was rarely mentioned as a drawback. This may be because of the selection of generally successful cases, many of which addressed health inequities. Each case showed evidence of addressing some, if not all, Q-Aims, though emphasis varied. For example, outreach cases improved patient care of at-risk groups while reducing risk of infectious disease spread in the population.

Fundamentally, the a priori assumption was that PC and PH collaboration can result in better outcomes. Synergistic opportunities were identified across cases that supported each organization’s aims. Collaborations between PC and PH brought together strengths from each partner. For example, PH built on pre-established community relationships with PC providers to deliver services to ‘hard to reach’ mothers, thereby addressing patient experience and population health. PC benefited from PH improvements to information systems aimed to manage vaccine information, while both took advantage of a coordinated immunization program to meet community demands and address population health goals.

Perceived successes in meeting some or all Q-Aims were observed in all cases. However, researchers and policy makers continue to caution against a too restrictive merger, should PH’s role in population health, health promotion and disease prevention erode under an integration model in which PH is pressured to relinquish resources to support PC clinical functions [1, 34, 35]. The models of collaboration offered here are not conclusive in this regard. Perhaps the message is to consider more appropriately other influences in which PC-PH collaborations are formed and operate. Researchers on this team have developed an ecological framework depicting different levels of influence (intra-personal, intra-personal, organizational, and systemic) on PC-PH collaborations [36–39]. Barriers and enablers to collaboration organized under these levels of influence were identified in the cases presented and align with those identified in our ecological framework thereby providing further evidence to support the framework. To what degree these factors impact the ability of collaborations to achieve all four Q-Aims is a question for future research, particularly in relation to population health and equity given concerns raised by PH advocates.

More recently, Q-Aim proponents are coming to terms with the concept of equity as fundamental to all constructs of the framework [40]. Application of an equity lens, most often claimed within a population health approach, pushes health systems to consider: how to make health care services accessible to marginalized populations within the context of universalism; how and whom to fund to deliver these services; how to support those best able to deliver these services; and how to share responsibilities with other sectors in an upstream, population health model recognizing broader determinants of health [23]. Arguments to support a focus on social justice and equity in primary health care have been made since the Declaration of Alma Ata [41] in 1978 and reiterated in the Ottawa Charter for Health Promotion [42] in 1986. However, these concepts are not explicitly communicated in the Q-Aims and as such risk being ignored or given less emphasis. Worse, the population health aim may be interpreted as the creation of universal programs that do not address the needs of sub-groups in the population that experience health inequities. The fear is that this can create further inequities. The problem is that improving population heath may also be interpreted as targeting the health of those population subgroups that are less healthy or do not have access to services [43, 44]. However, the definition of population health in Canada states that it is:

“an approach to health that aims to improve the health of the entire population and to reduce health inequities among population groups. In order to reach these objectives, it looks at and acts upon the broad range of factors and conditions that have a strong influence on our health” [45].

This definition invokes the concept of proportionate universalism [46] in which actions to reduce health inequities must be “universal but with a scale and intensity that is proportionate to the level of disadvantage” [47](p. 15). This study provided examples of collaborations in which practitioners passionately emulated and valued an equity and social justice view that was a driver to increase access to services for vulnerable populations. This requires willingness among those with high stakes in existing health system models to be open to alternatives for government investment to address inequities. Berwick and colleagues [17] argue for a balance in the Triple Aim, with the promise of equity:

“Gain in health in one subpopulation ought not to be achieved at the expense of another subpopulation. But that decision lies in the realms of ethics and policy; it is not technically inherent in the Triple Aim.” (p.760)

We agree but would add that it cannot be achieved at the expense of the health of the population as a whole. Thus, we argue that the goals of PC and PH to ensure health equity can move them farther forward in achieving the Q-Aim. Although health equity is included in the definition of population health, including it as a separate fifth aim would ensure that it receives the appropriate attention it deserves.

We selected cases that were brought to our attention through reports and key informants involved in a qualitative study [37–39]. Collaborations with less positive experiences could have been included although these were difficult to identify. Furthermore, we missed important insights from recipients of services. These need to be captured in future research, particularly given the Q-Aim of improving patient experience. Case studies captured participants’ perceptions of outcomes of collaboration rather than measured outcomes. Overall, more research is needed to examine critical outcomes of PC and PH collaborations, in particular the reduction of health inequities and cost analysis to further the understanding of financial impacts.

## Conclusion

PC and PH collaborations were created by PH and PC providers interested in the same community members, with mutual and/or compatible goals, and, who recognized through discussion and shared planning, advantages in forming relationships with each other. Recommended pre-conditions of the Q-Aim framework are worth noting – having a population health focus, operating within a learning system, and intersectoral collaboration with an ‘integrator role’, that, according to Whittington et al. [23] is best achieved as a shared function among participating agencies. These pre-conditions appeared to be true for the cases we investigated, acknowledging that agencies in some cases did take on a coordinator role. Continuing to appreciate local nuances, however, requires policies that enable local knowledge, relationships and circumstances to influence how collaborations adapt to specific community needs, as well as the patients and providers involved. A focus on health equity was integral in a number of cases. Equity is essential for transforming an integrated community-based primary care system and ought to be considered as a fifth aim.

## Supplementary information

**Additional file 1.** Intro Focus Group – For Moderators: Interview Guide for Front line Staff and Managers and Directors. This file contains the moderator’s guide for the first focus group for front line staff, and managers and directors.

**Additional file 2.** Moderator’s Focus Group Guide for the PSAT Follow Up for Front Line Staff, Managers and Directors. This file contains the moderator’s guide for the follow up focus group for front line staff, and managers and directors. This guide is focused on the concepts covered in the PSAT tool [31].

**Additional file 3.** Summaries of Ten Case Studies of Primary Care and Public Health Collaboration. This file contains details of each case such as the context, partners involved, health issues addressed, precipitators of the collaboration, problem being addressed by the collaboration goals, key factors influencing success of the collaboration, community involvement in the collaboration, and key impacts and outcomes.

## Data Availability

The datasets generated and/or analysed during the current study are not publicly available due to restrictions from ethics but aggregated data are available from the corresponding author on reasonable request. Ethics board approval would be required to obtain the data.

## Abbreviations

MD: Medical doctor
OT: Occupational Therapist
PC: Primary Care
PH: Public Health
PHN: Public Health Nurse
PSAT: Partnership Self-Assessment Tool
Q-Aim: Quadruple Aim
RN: Registered Nurse
