# Supplementary material for: Addressing quadruple aims through primary care and public health collaboration: ten Canadian case studies
Source: BMC Public Health. 2020 Apr 16;20:507. doi: 10.1186/s12889-020-08610-y (PMC7164182; doi:10.1186/s12889-020-08610-y)
Supplement: Supplementary file 1 — Additional file 1. Intro Focus Group – For Moderators: Interview Guide for Front line Staff and Managers and Directors. This file contains the moderator’s guide for the first focus group for front line staff, and managers and directors. [file 12889_2020_8610_MOESM1_ESM.docx]

**INTRO FOCUS GROUP - FOR MODERATORS**

**Interview Guide for Front line Staff and Managers and Directors**

**DEMOGRAPHICS:** Before we begin, I want to remind you that you do not have to answer any questions that you do not feel comfortable with. Also, as we begin the focus group please introduce yourself by your **first name** and tell us your **discipline**, which **sector** you represent (primary care, public health or other), and how many **years you have been working in your discipline and this 'sector'**. We will be changing your name on the transcript to a fictitious name and we will be sure to protect your confidentiality if we use any quotes in any reporting of results.

****Support for Innovation**

1. What programs/services are delivered in this collaboration and how are they delivered?
2. Describe in general how you work together in your collaboration. (*Prompts:* how do you respond to needed adjustments?)
3. What is different about how you deliver services to this population now compared to before this collaboration existed?
4. What drove the development of this collaboration? (*Prompts:* individual interests; local, regional, provincial systems level issues or changes)

****Goals**

1. What do you think are the goals of this collaboration?
2. How were goals defined? (*Prompts:* Who was involved? i.e. front line practitioners, MoH, physicians, community members?)
3. Have the goals been reached? If yes, how so and if not – why not?

****Client/Community Engagement**

1. What impact did primary care players, public health players, clients/patients/community members and/or other organizations each have on the development of goals for this collaboration?

****Information Exchange**

1. Describe information structures or processes that facilitate or create barriers for exchange of information between partners in your collaboration? (*Prompts:* communication tools, rounds, EMRs, email, team meetings) (*Prompts:* patient’s/client’s information privacy concerns, if any exist? How do you stay connected?)
2. What type of information is shared between individuals in the organizations as well as between organizations in the collaboration?
3. How do you share feedback between collaboration partners? (*Prompts:* How do these mechanisms work?)

****Mutual acquaintanceship**

1. (Managers/directors only) How were roles and scope of practice of various primary care and public health players in the collaboration determined?
2. (Front line only) How was your role or scope of practice in the collaboration determined?
3. Primary care and public health have different approaches to working with patients /clients/groups and populations. How do you think your approaches differ and how do these differences impact on your collaboration? (*Prompts if needed:* bio-medical, behavioural, socio-environmental approaches; downstream versus upstream; individual and family versus group and population focus)
4. Describe any activities that occur among people working in this collaboration that help to build knowledge of one another’s worlds? (*Prompts:* What activities occur? How often do they occur? How do you feel about these activities?)

****Formalization Tools**

1. For professionals participating in collaborations/partnerships, it can be important to know what is expected of you and what you can expect of others. What formalized tools exist, if any, that helped to clarify roles and activities in your collaboration and what do you think about them? (*Prompts:* protocols, job descriptions, agreements, information sharing systems)
   1. How were these tools created? By whom?

****Trust**

1. How has your relationship with staff members in your collaborative partner organizations changed since the collaboration began?
2. How do you think trust is built and maintained between partners in this collaboration?
3. What threatens trust in your collaboration and how do you deal with it?
4. How are conflicts in the collaboration addressed?

****Evaluation**

1. How many clients are reached through your collaborative programs and services?
2. What, if any, evaluations have been conducted of this collaboration?
   1. How often do such evaluations occur and what and who do they involve?

**Centrality**

1. Describe any formal structures and processes that support action to maintain and sustain the collaboration? Think about the organizational level and the larger systems level. (*Prompts:* formalized agreements; decision-making tools, regular team meetings)

**Financial and Non-Financial Resources**

1. What financial and non-financial resources facilitate or create barriers in your collaboration? (*Prompts:* Staff time, partnerships with other agencies or institutions; grants, skills and expertise)
2. How were you able to obtain these resources? What barriers did you have to overcome and how did you overcome them?
3. Are there any incentives (financial or other) tied to performance in the collaboration? (*Prompts* financial incentives for high screening rates). If so, what do you think about these incentives?

**Systems Factors**

1. Describe any system level factors that are outside of the organizations that have influenced this collaboration. (*Prompts:* provincial or municipal mandates, professional standards of practice, shared information systems, government restructuring, inter-professional education initiatives; national position papers, quality improvement initiatives; who are the key players in sustaining the collaboration?)
