# Supplementary material for: Addressing quadruple aims through primary care and public health collaboration: ten Canadian case studies
Source: BMC Public Health. 2020 Apr 16;20:507. doi: 10.1186/s12889-020-08610-y (PMC7164182; doi:10.1186/s12889-020-08610-y)
Supplement: Supplementary file 2 — Additional file 2. Moderator’s Focus Group Guide for the PSAT Follow Up for Front Line Staff, Managers and Directors. This file contains the moderator’s guide for the follow up focus group for front line staff, and managers and directors. This guide is focused on the concepts covered in the PSAT tool [31]. [file 12889_2020_8610_MOESM2_ESM.docx]

**Moderator’s Focus Group Guide for the PSAT Follow Up for Front Line Staff, Managers and Directors**

**DEMOGRAPHICS:**

Before we being, I want to remind you that you do not have to answer any questions that you do not feel comfortable with. Also, as we begin the focus group please introduce yourself by your

1. first name and
2. tell us your discipline and
3. number of years in this discipline. Finally,
4. which sector you represent (primary care, public health or other) and
5. how many years you have been working in this 'sector'.
6. Finally, it would be helpful if you would tell us if you have ever worked in the other sector.

We will be changing your name on the transcript to a fictitious name and we will be sure to protect your confidentiality if we use any quotes in any reporting of results.

The goal of this focus group is to get a deep understanding of the processes and structures involved in building and maintaining a strong collaboration? We will talk about a number of concepts that are thought to be integral to understanding collaboration. The first is synergy.

**SYNERGY**

Synergy occurs by combining the different kinds of knowledge, skills, and resources of its participants. It is this combining power than enables people and organizations in a collaboration to accomplish more than any of them can on their own.

1. **Thinking about the people and organizations in your collaboration, how well do you think your collaboration is building synergies?**

Consider things such as:

- Identifying new and creative ways to solve problems
- Including the views and priorities of the people affected by the partnerships work
- Developing goals that are widely understood and supported among partners
- Identifying how different services and programs in the community relate to other problems the partnership is trying to address
- Responding to the needs and problems of the community
- Implementing strategies that are most likely to work in the community
- Obtaining support from individuals and organizations in the community that can either block the partnership's plans or help move them forward
- Carrying out comprehensive activities that connect multiple services, programs, or systems
- Clearly communicating to people in the community how the partnership's actions will address problems that are important to them

1. **Why do you think your collaboration did well or not well in this area?**

**LEADERSHIP**

1. **Please think about all of the people who provide either formal or informal leadership in this partnership. How effective is leadership in this partnership?**

Consider things such as:

- Taking responsibility for the partnership
- Inspiring or motivating people involved in the partnership
- Empowering people involved in the partnership
- Communicating the vision of the partnership
- Working to develop a common language within the partnership
- Fostering respect, trust, inclusiveness, and openness in the partnership
- Creating an environment where differences of opinion can be voiced
- Resolving conflict among partners
- Combining the perspectives, resources, and skills of partners
- Helping the partnership be creative and look at things differently
- Recruiting diverse people and organizations into the partnership

1. **Why do you think your collaboration was effective or not effective in this area?**

**ADMINSTRATIVE AND MANAGEMENT**

1. **We would like you to think about the administrative and management activities in your collaboration. How effective is your collaboration in carrying out administrative and management activities?**

Consider things such as:

- Coordinating communication among partners, and with people and organizations outside the partnership
- Organizing partnership activities (meetings and projects)
- Applying for and managing grants and funds
- Preparing materials that inform partners and help them make timely decisions
- Performing secretarial duties
- Providing orientation to new partners as they join the partnership
- Evaluating the partnership (progress and impact)
- Minimizing barriers to participation in partnership meetings and activities (e.g., by holding them at convenient places and times, and by providing transportation and childcare)

1. **Why do you think your collaboration was effective or not effective in this area?**

**NON FINANCIAL RESOURCES**

1. **How efficiently and effectively does your collaboration use partners’ non-financial resources including in-kind resources and partners’ time to achieve the collaboration’s goals?**

Consider things such as:

- Skills and expertise (e.g., leadership, administration, evaluation, law, public policy, cultural competency, training, community organizing)
- Data and information (e.g., statistical data, information about community perceptions, values, resources, and politics)
- Connections to target populations, political decision-makers, government agencies, other organizations/groups
- Legitimacy and credibility
- Influence and ability to bring people together for meetings and activities

1. **Why do you think your collaboration did well or not well in this area?**

**FINANCIAL AND OTHER CAPITAL RESOURCES**

1. **How efficiently and effectively does your collaboration use financial and other capital resources to achieve the collaboration’s goals?**

Consider things such as:

- Money
- Space
- Equipment and goods

1. **Why do you think your collaboration did well or not well in this area?**

**DECISION MAKING**

1. **How well do you think decision making works in your collaboration?**

Consider things such as:

- Comfort with the way decisions are made
- Frequency that you support decisions made by the collaboration
- Feeling left out of the decision making process

1. **Why do you think your collaboration did well or not well in this area?**

**BENEFITS OF COLLABORATION**

1. **A result of your participation in the collaboration, what benefits have you experienced?**

Consider things such as:

- Enhanced ability to address an important issue
- Development of new skills
- Heightened public profile
- Increased utilization of my expertise or services
- Acquisition of useful knowledge about services, programs, or people in the community
- Enhanced ability to affect public policy
- Development of valuable relationships
- Enhanced ability to meet the needs of my constituency or clients
- Ability to have a greater impact than I could have on my own
- Ability to make a contribution to the community
- Acquisition of additional financial support

1. **Why do you think you experienced these benefits?**

**DRAWBACKS OF COLLABORATION**

1. **A result of your participation in the collaboration, what drawbacks have you experienced?**

Consider things such as:

- Diversion of time and resources away from other priorities or obligations
- Insufficient influence in partnership activities
- Viewed negatively due to association with other partners or the partnership
- Frustration or aggravation
- Insufficient credit given to me for contributing to the accomplishments of the partnership
- Conflict between my job and the partnership's work

1. **Why do you think you experienced these drawbacks?**

**COMPARING BENEFITS AND DRAWBACKS**

1. **So far, how have the benefits of participation in this collaboration compared to the drawbacks?**

**SATISFACTION WITH PARTICIPATION**

1. **How satisfied are you in this collaboration?**

Consider things such as:

- the way the people and organizations in the partnership work together
- your influence in the partnership
- your role in the partnership
- the partnership’s plans for achieving its goals
- the way the partnership is implementing its plans

1. **Are there any other comments that you would like to share?**

**Thank you!**
