# Supplementary material for: Addressing quadruple aims through primary care and public health collaboration: ten Canadian case studies
Source: BMC Public Health. 2020 Apr 16;20:507. doi: 10.1186/s12889-020-08610-y (PMC7164182; doi:10.1186/s12889-020-08610-y)
Supplement: Supplementary file 3 — Additional file 3. Summaries of Ten Case Studies of Primary Care and Public Health Collaboration. This file contains details of each case such as the context, partners involved, health issues addressed, precipitators of the collaboration, problem being addressed by the collaboration goals, key factors influencing success of the collaboration, community involvement in the collaboration, and key impacts and outcomes. [file 12889_2020_8610_MOESM3_ESM.docx]

# Summaries of Ten Case Studies of Primary Care and Public Health Collaboration

NOTE: In the tables below, we use the term health authority/authorities to refer generically to Local Health Integration Networks, District Health Authorities and Regional Health Authorities.

| Table 1: Primary Care and Public Health Collaboration: Cases Related to Provider Capacity Building | | |
| --- | --- | --- |
| PC= Primary Care  PH= Public Health | **CASE I: Enhanced Well Baby Visits** | **CASE 2: Comprehensive Tobacco Cessation** |
| **Context** | Large urban centre | Northern rural community |
| **Partners involved in Collaboration** at time of study and main activity of partners | **Primary Care**:   - Interdisciplinary PC team with satellite offices   **Public Health**:   - Family health program Public Health Nurse (PHN) seconded to PC part-time - PHN seconded to work with PC practitioners and worked with nurses and physicians to teach Rourke and Nipissing screening tools to enhance the 18 month well baby visit - PHN constantly shared resources, both community, print, and media with PC | **Primary Care (PC)**:   - Interdisciplinary PC team with satellite offices   **Public Health (PH)**:   - Public Health Nurse (PHN) on Tobacco Team   **Other**   - Hospital and other community agencies involved with PC and PH in a regional network to work on socio-environmental issues to reduce smoking (by-laws re smoking in public places) |
| **Health Issues Addressed** | - Enhanced well baby visits including assessment and screening for developmental delays | - Smoking Cessation |
| **Precipitators of collaboration** | - Historically PH had good relationship with PC, however in the recent past it deteriorated and PH wanted to improve their relationships with PC - PH thought the 18 month well baby visit was a good project for collaboration as they could offer resources and expertise to enhance PC practice - The Interdisciplinary PC team wanted to work closely with community and thought child health would be a good venue | - High smoking rates in region - PH unit strategic plan to work more closely with PC in region - PC proposal submitted with plan for smoking cessation program; saw PH as potential partner (NP had previously worked in PHU) - Local organizations formed tobacco network for action on smoking in region |
| **Problem being addressed by Collaboration** | - Lack of consistency in doing the 18 month well baby visit - Limited information and resources related to 18 month well-baby for parents attending PC interdisciplinary team | - Among highest smoking rates in the province - Region lacked smoking cessation programming - PC desire for capacity building related to evidence-based tobacco cessation programming smoking |
| **Goals** | - Build capacity of PC staff for enhanced 18 month well baby visit - Strengthen partnerships between PH and PC - Achieve consistent messaging for patients - Document, intervene with children with developmental delay | - Increase application of tobacco cessation best practice among PC providers - Reduce smoking rates in region |
| **Key Factors influencing success of the collaboration** | **INTRAPERSONAL**  **PERSONAL QUALITIES, KNOWLEDGE AND SKILLS**  - PHN valued by PC staff as extremely knowledgeable  **INTERPERSONAL**  **ROLE CLARITY**   - Role established early in collaboration although not communicated to all players   **EFFECTIVE COMMUNICATION**   - Lack of communication experienced among front line PC physicians about the collaboration; busy schedules added to communication problems - PC nurses were meeting with PHN regularly and did not share this problem   **TRUSTING AND INCLUSIVE RELATIONSHIPS**   - Very high levels of trust and extensive areas of common turf felt between PC nurses and PHN   **ORGANIZATIONAL**  **Clear Mandates, Vision and Goals for collaboration**   - A formal contractual agreement (MOU) developed between PC and PH   **strategic Coordination and Communication Mechanisms between partners**   - MDs perceived they were not part of the development of the collaboration, therefore were not bought in or in the know about it; "we are going from the top to the bottom instead of in reverse"   **formal organizational Leaders as collaboration Champions**   - PC and PH leaders at administrative level supported the collaboration   **COLLABORATIVE ORGANIZATIONAL CULTURE**   - PC nurses expressed concerns that they were conducting the 18 month well baby visit, but physicians were receiving payment for it particularly since they were not compensated for their time to obtain training for this enhanced skill - PC practice is extremely busy and therefore it was challenging find time for the collaboration   **OPTIMAL USE OF RESOURCES**   - Moderate to extensive sharing of resources re well child assessment and information about community resources   **OPTIMAL USE OF HUMAN RESOURCES**   - PC purchased services of PHN through a secondment; funding 50% position for large Interdisciplinary PC team with satellites - Time was a huge challenge for PC physicians; different time constraints affecting physicians' ability to implement a collaboration. Also challenging to add another practice expectation regarding an enhanced 18 month assessment to a busy PC workload - Inadequate funding for training; PC nurses not supported by PC organization for training activities; done on personal time - H1N1 outbreak diverted collaboration activities   **COLLABORATIVE APPROACHES TO Programs and Services Delivery**   - Interdisciplinary PC team works as interdisciplinary teams | **Organizational**  **Clear Mandates, Vision and Goals for collaboration**   - Clear mandate and congruent focus by all partners to work on smoking cessation - Evolution of the collaboration over 4 years led to new staff lacking knowledge of history goals of collaboration and updates needed   **TRUSTING AND INCLUSIVE RELATIONSHIPS**   - Past and existing strong relationships between NP and PHN who were key in the collaboration - Past NP experience in other sector helped increase understanding of the other sector   **ROLE CLARITY**   - Clear roles established early and reviewed regularly (through terms of reference) - Specific PHN assigned to work on collaboration   **OPTIMAL USE OF RESOURCES**   - Sharing of resources (e.g., supplies, informational resources) highly valued by partners - PHN knowledge re funding sources to support programming highly valued by PC - Shared lunch room space greatly facilitated strengthening staff relationships; trust and commitment to collaboration supported   **OPTIMAL USE OF HUMAN RESOURCES**   - PHN assigned to tobacco team and to work with PC - H1N1 diverted collaboration activities   **COLLABORATIVE ORGANIZATIONAL CULTURE**   - PC nominated for a tobacco cessation award and acknowledgment of excellence by Medical Office of Health demonstrated valuing of PC work - Joint training sessions strengthened staff relationships   **SYSTEMIC**  **GOVERNMENTAL and REGULATORY POLICIES and MANDATES FOR COLLABORATION**   - Provincial standards provided mandate for PHU to work in partnership with partners - PH Mandates from Ministry for tobacco reduction work a key driver for collaboration - national and provincial tobacco reports enhanced team readiness for collaboration on cessation agenda   **FUNDING MODELS AND FINANCIAL INCENTIVES SUPPORTING COLLABORATION**   - Interdisciplinary PC team Ministry mandates for building health promotion programming in care - Smoke Free Ontario mandates and supports for training key enabler - Inequities existed with regard to which nurses were supported to attend tobacco cessation training; RNs not paid through specialized PC funding envelope were not eligible to attend |
| **Community Involvement in collaboration** | - Initial goals were based on community needs, but not community input - Other community programs (i.e. literacy programs) informally joined in once they saw the collaboration in action | - No community involvement from community members except for feedback collected from attendees of group tobacco cessation sessions |
| **Key impacts and Outcomes** | - Increased consistency implementing 18 month well baby visit - Improved thoroughness implementing 18 month well baby visit - Increased parent satisfaction - Increased PC nurses competency (enhanced skills) and working to full scope of practice | - Perceived successful capacity building   - PC staff able to run programs independently   - PC nurses noted increased confidence in skills gained from guidance/mentorship provided by PHN - Perceived increase in evidence-base practice behaviours in PC regarding tobacco reduction services - Increased access to variety of materials and supplies in PC (NRTs, dental supplies, literature, etc.) - Too soon to say if smoking rates have changed in region; study currently underway to explore this - PC perceives program has worked to increase smoking reduction among patients in practice - Spin offs from the collaboration - access to dental care supplies - considering other programs for collaboration - PHN can bring resources developed from this collaboration to other communities/practices (Medical Directives, planning materials) |
| **Research and/or Evaluation** | - Evaluation of collaboration was conducted earlier but not shared with partners at all levels | - PC bought into continuous quality improvement which helped build trust, strengthened relationships and the partnership - Consistent informal feedback shared by members at every meeting |
| **Other important notes** | - Case study acted as an intervention. Some PC physicians were not informed about background and goals of the collaboration PH recognized importance of involving front line providers when collaboration expands to include other sites or with staffing changes | - Currently in a stage of renewal; case study acted as a intervention to prompt renewal process - Gap in perspectives as no physician participation in study |

| Table 2: Primary Care and Public Health Collaboration: Cases focusing on Regional Vaccine/ Immunization Management | | |
| --- | --- | --- |
| PC= Primary Care  PH= Public Health | **CASE 3: Regional E-Health for Immunization Management** | **CASE 4: Vaccine Management and Information Exchange** |
| **Environmental context** | Northern urban / rural community | Urban and rural community |
| **Partners involved in Collaboration *at time of study*** | **Primary Care:**   - Health Centre serves most of the population in the region   **Public Health:**   - PH organization serving region - Staffing clerk hired to coordinate flu clinics for both organizations; appointments for immunizations made by phone through booking clerks   **Other Partners:**   - Worked with local hospital electronic documentation system | **Primary Care:**   - Recently opened PC office with multidisciplinary team   **Public Health:**   - PH organization serving region - Staff from communicable disease program involved   **Other Partners:**   - Medical Officer of Health/Medical Health Officer offered participation in collaboration to all PC practices in region; almost all agreed to participate (one PC practice included in case study) |
| **Health Issues Addressed** | - Immunizations (Flu clinics was focus although involved other immunizations and development and implementation of EHR for immunizations for region) | - Vaccine immunization program [Exchange of Information and Resources pertaining to Vaccine and Immunization Records] |
| **Precipitators of collaboration** | - Long line ups experienced in past for flu vaccines - National negative media coverage of above and community dissatisfaction caused concerns for partners - PC and PH working with the same populations so need to collaborate seemed logical decision | - PH not collecting immunization records. [Current legislation states it is a parental responsibility to report their child's immunizations to PH] - PC physicians finding management of vaccines onerous and time-consuming; collaborative initiative between PC and PH incorporated the following:-   - PH delivers monthly vaccine order to PC’s   - PH concurrently checks cold chain log sheet (faxing no longer required).   - PH restocks depleted vaccine supplies as needed   - PH shares flu vaccine record from PH clinics with PC. PC reports immunization data administered to children 18 years and under, PH to add to provincial database - Exchange of immunization data could result in:   - greater accuracy and completeness of immunization data between PC PH and province   - less telephone contacts between PC, PH and parents, particularly during the PH's annual legislated school suspension process for students without up-to-date immunizations |
| **Problem being addressed by Collaboration** | - Long wait times for flu vaccines (H1N1) and lack of shared records of immunizations between PC and PH | - Inefficiencies of PH’s reporting of school age immunization records and PC’s management of the vaccines (cold chain, etc.) |
| **Goals** | - Reducing wait times for flu immunization | - Collaborative initiative between PC and PH to increase efficiencies of school age children and youth immunization reporting to Ministry and PC’s management of vaccines |
| **Key Factors influencing success of the collaboration** | **INTERPERSONAL**  **TRUSTING AND INCLUSIVE RELATIONSHIPS**   - Partners had strong past trusting relationships; small community and many previous working relationships - Front line staff from both organizations had positive relationships with each other and saw opportunities to solve the problem   **ORGANIZATIONAL**  **Clear Mandates, Vision and Goals for collaboration**   - Formal agreements clarified issues of: privacy, ownership of Electronic Health Record (EHR) IT systems and data, responsibilities and accountabilities - PC and PH leadership had strong positive past relationships partly influenced by community size - Shared common vision from top leaders to front line in PC and PH   **strategic Coordination and**  **Communication Mechanisms between partners**   - Standardized training on the EHR system occurred with nursing staff in PC and PH - Some difficulties in communicating vision to all partners as time dedicated to meeting service delivery needs rather than needs of collaborative partners   **formal organizational Leaders as collaboration Champions**   - Leadership of both organizations had common vision to address problem - Changing leadership over time and being clear about who leaders are challenging   **COLLABORATIVE ORGANIZATIONAL CULTURE**   - PC and PH's collaborative versus competitive beliefs and culture   **OPTIMAL USE OF RESOURCES**   - In future, partnership required to update the IT system requiring significant resources - IT system was considered a legacy system and needs upgrading creating challenge; this introduces a new phase in collaboration   **OPTIMAL USE OF HUMAN RESOURCES**   - Each organization leveraged its strengths: - PH leveraged PC's presence in community for delivery of flu clinics through PC - Community volunteers supported smooth and efficient clinic functioning   **COLLABORATIVE APPROACHES TO Programs and Services Delivery**   - Integrated program of immunization service delivery with PC and PH - No community involvement in the planning of the collaboration although feedback sought from volunteers for level of satisfaction   **SYSTEMIC**  **HARMONIZED INFORMATION AND COMMUNICATION INFRASTRUCTURE**   - IT upgrades could greatly benefit from provincial/ national infrastructure support; the region has offered suggestions to the province on ways to design and implement a system between sectors | **INTRAPERSONAL**  **PERSONAL QUALITIES, KNOWLEDGE AND SKILLS**  - Positive Personal Characteristics of Practitioners   - PH Delivery person was front line “face” of PH - Positive personality supports relationship building - Experience and knowledge facilitates collaboration - Past work experien ce of RNs in both sectors meant better knowledge base   **PERSONAL VALUES , BELIEFS AND ATTITUDES**   - Professionals who did not support immunizations had little impact on collaboration's success   **INTERPERSONAL**  **ROLE CLARITY**   - Identified role and scope of players had positive effect   **EFFECTIVE COMMUNICATION**   - Using effective communication strategies (phone, email, face-to-face and regular contact) had positive effect   **TRUSTING AND INCLUSIVE RELATIONSHIPS**   - Strategies used for developing and maintaining positive relationships   - Despite PH’s “enforcement role” related to cold chain management and school suspensions, PHN built strong relationships with PC providers to avoid “adversarial feelings” - Relationship building took time   **ORGANIZATIONAL**  **strategic Coordination and Communication Mechanisms between partners**   - Processes and strategies influencing success included: formal letters, emails, meetings - Effective and efficient client-related information sharing was key - Consultation with College of Physician and Surgeons helpful   **Clear Mandates, Vision and Goals for collaboration**   - PH strategies to 'sell collaboration' and vaccine immunization program to the public and making collaboration appealing to increase partner buy-in (framed as increasing PC efficiencies and reducing costs)   **formal organizational Leaders as collaboration Champions**   - Strong representation of leadership by PH: MOH proposed collaboration between PH & PC as a “win-win" collaboration   **COLLABORATIVE ORGANIZATIONAL CULTURE**   - Effective conflict management processes included PH sending letters to address PC vaccine management problems; follow-up with phone calls; PH admits importance about being selective regarding issues to address; positive approach important   **OPTIMAL USE OF RESOURCES**   - Funding issues and sharing resources to support the collaboration   **OPTIMAL USE OF HUMAN RESOURCES**   - Human resource capacity enhanced when RNs work in both sectors - Nurses in PH assigned to work with specific practices   **COLLABORATIVE APPROACHES TO Programs and Services Delivery**   - PH – PC collaboration is in transition in region and more work to be done to consider more ways to improve processes - No direct community involvement in collaboration - Community benefited from efficient vaccine immunization program |
| **Community Involvement in collaboration** | - No community involvement from community members except for community volunteers to work in flu clinics | - No community involvement from community members except for informing them about sharing immunization data between PC and PH through use of flyers (e.g., in PC offices) |
| **Key impacts and Outcomes** | - No line ups for flu vaccines - Common EHR implemented for PC and PH for immunization tracking - Improved capacity of PC and PH nursing staff related to accessing immunization history using common EHRs - Reduced school suspensions due to more complete immunization records | - Reduced vaccine wastage costs - Enhanced patient experience related to improved work processes; everything runs smoother; direct PC PH communication - Improved PC and PH access to vaccine and immunization data - Reduced per capita cost; cost saving resource allocation - Clients directly benefit from collaboration with improved PC vaccine management program and PH reporting of immunization of children under 18 years of age to Ministry; consequently, reduced school suspensions due to more up-to-date immunizations and all have accurate records |
| **Evaluation and/or Research** | - Testing of EHR system occurred at various levels and with end users demonstrating evaluation was conducted - Evaluations each year occurred with staff and volunteers who used flu clinics to look for improvements | - No formal evaluation although statistics kept for vaccine wastage as reported to provincial Ministry |

| Table 3: Primary Care and Public Health Collaboration: Cases Related to Community-based Health Promotion Programming | | | |
| --- | --- | --- | --- |
| PC= Primary Care  PH= Public Health | **CASE 5: Rural Community Health Initiative** | **CASE 6: Women's Health** | **CASE 7: Rural Health Promotion** |
| **Environmental context** | - Small rural community | - Rural community | Rural community |
| **Partners involved in Collaboration *at time of study*** | **Primary Care (PC):**   - PC physicians   **Public Health (PH):**   - Ministry; health authority   **Other partners:**   - Researchers; community-based steering committee; local and regional, government sectors (health and social services Ministries), NGOs, First Nations communities, parks and recreation | **Primary Care:**   - Physician, Nurse Practitioner   **Public Health:**   - PHNs, nutritionists, public health managers - Partnership agreement with a PC physician, an NGO, health authority   **Other partners:**   - community representatives, a midwife, and coordinator participate in program planning and delivery | **Primary care**   - NPs, RNs, LPNs, family physicians, managers, administrators   **Public Health**   - PHNs, nutritionists, youth coordinators, health educators, public health managers, administrators   **Other partners:**   - School board, mental health, addictions services, community representatives, and family centre |
| **Health Issues Addressed** | - Youth health, mental health and addictions, food security and social determinants of health | - Women’s health | - Well baby, child and youth health issues |
| **Precipitators of collaboration** | - Lack of mental health services in community - Common frustration felt in community related to problems - Had a common vision to address problem - Awareness of need to collaborate and to do things differently | - Community need for better services and access to health services for adolescent and adult women. - Source of funding available when all partners came together - Partners shared common vision | - Leader with PC PH experience had vision of the potential impact of collaboration between PH and PC - Community concern regarding well baby and youth health - Concern about access to care for families without a family doctor |
| **Problem being addressed by Collaboration** | - Region lacked mental health and addiction services; need to address barriers in accessing services and health care - Difficult for clients to navigate through the healthcare system - Absence of local data on mental health and addiction services | - Adolescent and adult women face barriers to accessing confidential and comprehensive health care in this rural community - Women in outlying communities face additional barriers such as finding culturally relevant care, and availability of transportation | - Health authority administrator saw way to reduce service duplication - Well baby collaboration; PC trained PHNs in Rourke assessment: Clients without family doctor assessed - Youth health centre initiated to address youth alcohol abuse and mental health issues in community - Lack of access to PC providers and youth-specific health services - Immunization programs inefficient |
| **Goals** | - Provide timely and low-threshold access to mental health and addiction services - Create seamless network connecting PC, community-based providers, and formal mental health system - Improve care processes - Conduct local data collection/analysis - Reduce health inequalities | - Provide women-centred primary health care to underserved women in the community | - Improve comprehensiveness of well child and youth health care - Reduce duplication in immunization programs |
| **Key factors influencing success of collaboration** | **INTRAPERSONAL**  **PERSONAL QUALITIES, KNOWLEDGE AND SKILLS**   - People interested in specific projects; not committed to entire collaboration - Individuals' particularly useful skill sets - Many devoted personal time; key people needed to maintain collaboration   **PERSONAL VALUES , BELIEFS AND ATTITUDES**   - Aware of need/ willingness to collaborate   **INTERPERSONAL**  **ROLE CLARITY**   - Roles flexible; individuals with particular strengths took corresponding roles   **TRUSTING AND INCLUSIVE RELATIONSHIPS**   - Being committed built trust - Volunteer appreciation shown - Knowing players; regular interactions   **SHARED VALUES, BELIEFS, AND ATTITUDES**   - Addressed challenges in non-confrontational manner; open attitudes; valued transparency and accountability   **EFFECTIVE CLINICAL DECISION PROCESSES**   - PC MD felt left out of decision-making   **ORGANIZATIONAL**  **CLEAR MANDATES, VISION AND GOALS FOR COLLABORATION**   - Varying organizational goals/philosophies - Developed strategic plan, goals, terms of reference - Informal structure left collaboration vulnerable   **STRATEGIC COORDINATION and COMMUNICATION MECHANISMS BETWEEN PARTNERS**   - Communication processes differed - Time/geography challenge organizing meetings; monthly meetings for providers - Leadership changes negatively impacted relationships/connections   **formal organizational Leaders as collaboration Champions**   - Formal organizational leaders bought in; collaboration champions   **COLLABORATIVE ORGANIZATIONAL CULTURE**   - Informal, round table, consensus process - No conflict resolution protocols - Organizational readiness to break silos   **OPTIMAL USE OF RESOURCES**   - Resources decreased; inadequate time allocated for collaborative efforts - Had funding from various sources and meeting space   **OPTIMAL USE OF HUMAN RESOURCES**   - Insufficient staff for population needs   **COLLABORATIVE APPROACHES TO** **PROGRAMS AND SERVICES DELIVERY**   - Partnership complex; organizations geographically dispersed - Flexible, client-centered services   **SYSTEMIC**  **GOVERNMENTAL AND REGULATORY POLICIES AND MANDATES FOR COLLABORATION**   - Top-down mandate supported collaboration; no idea how to implement - Changing policies and structures to allow collaboration challenging   **FUNDING MODELS AND FINANCIAL INCENTIVES SUPPORTING COLLABORATION**   - Competition for scarce resources; momentum dependent on financing   **HEALTH SERVICE STRUCTURES THAT** **PROMOTE COLLABORATION**   - Political changes affected collaboration, i.e., provincial changes in PC services | **INTRAPERSONAL**  **PERSONAL VALUES , BELIEFS AND ATTITUDES**   - Those involved in collaboration believe in women-centred care   **INTERPERSONAL**  **ROLE CLARITY**   - Key challenge was trying to find a good fit for PH personnel in a mostly PC setting   **TRUSTING AND INCLUSIVE RELATIONSHIPS**   - Commitment shown to the collaboration built trust among partners   **SHARED VALUES, BELIEFS, AND ATTITUDES**   - Committed staff who bought into philosophy of women-centred care   **ORGANIZATIONAL**  **Clear Mandates, Vision and Goals for collaboration**   - Formal written operational plans that promote or enhance cross-sectoral planning, coordination and implementation   **strategic Coordination and Communication Mechanisms between partners**   - Collaboration has several committees composed of health team and community members - Committee meetings and formal agreements helped enhance communication, build relationships, encourage participation in decision-making, and created a sense of accountability among partners   **formal organizational Leaders as collaboration Champions**   - The right mix of leaders with a similar vision. PH and PC provided guidance in strategic planning for the collaboration   **RESOURCES**   - Funding limited; remaining resources come from in-kind contributions from participating agencies, i.e. space and staff time - High staff turnover rates and limited time for orientation of new staff to the collaboration   **SYSTEMIC**  **GOVERNMENTAL AND REGULATORY POLICIES AND MANDATES FOR COLLABORATION**   - PH at the provincial level undergoing major changes resulting in some role confusion at provider level | **INTERPERSONAL**  **ROLE CLARITY**   - PH providers are unsure of their role in the collaboration   **TRUSTING AND INCLUSIVE RELATIONSHIPS**   - Joint training and meetings increased trust and knowledge of PC and PH services   **ORGANIZATIONAL**  **Mandates, VisionS and Goals for collaboration**   - Conflicting mandates between PC and PH impeded collaboration   **strategic Coordination and**  **Communication Mechanisms between partners**   - PH-PC meetings enabled opportunity for role negotiation   **formal organizational Leaders as collaboration Champions**   - Formal organizational leader drove collaboration forward   **RESOURCES**   - Demands on PC providers diverted time from collaboration with PH - Resources needed to share responsibility of leading the collaboration   **Health Human RESOURCES**   - High turnover in personnel interrupted relationships and communication flow - No orientation process to PC PH collaboration for new employees   **SYSTEMIC**  **GOVERNMENTAL AND REGULATORY POLICIES AND MANDATES FOR COLLABORATION**   - PH at the provincial level was undergoing major changes resulting in some role confusion at the provider level |
| **Community Involvement in collaboration** | - Community-based steering group designed, directed, supported services - Community guiding principles to ensure client-centered service and transparent communication - Information shared with all members | - Community representatives sat on committees - Community connections used to understand local women’s needs and develop responsive programming | - Community representatives involved in needs assessment   and program development |
| **Key impacts and Outcomes** | - Provided one-stop shop for patients - Improved access to PHC for youth - Developed web resource inventory - Established community foundation steering group that provided a strong voice for the community - Enhanced communication/improved relationships between partners - Fostered community engagement built on local capacity - Developed new projects | - Underserved women gained better access to health services - Women with complex needs managed better in a multidisciplinary setting - Strong relationships formed among partners, enhancing trust and commitment to collaboration | - Improved accessibility and comprehensiveness of health promotion and illness prevention health services for children/youth - Joint PH/PC flu clinics reduces duplication of services, increases efficient delivery of immunizations, increases client satisfaction - New relationships formed among PH/PC partners, enhancing future   communication and collaboration |
| **Evaluation and/or Research** | - Satisfaction surveys were conducted - Evaluation matrix was conducted - No formal evaluation processes | - Currently developing an evaluation framework - No formal evaluation done to date | - No formal evaluation done to date |

| Table 4: Primary Care and Public Health Collaboration: Cases Focusing on Increasing Access to Care through Outreach Programs and Services for Vulnerable Populations | | | |
| --- | --- | --- | --- |
| PC= Primary Care  PH= Public Health | **CASE 8: Urban Child Health Promotion and Family Outreach** | **CASE 9: Inner City Outreach** | **CASE 10: Street Health Outreach** |
| **Environmental context** | Large urban center | Urban Centre | Urban |
| **Partners involved in Collaboration *at time of study*** | **Primary Care (PC):**   - PC services, PC physician   **Public Health (PH):**   - Health authority; NPs hired by the health authority   **Other Partners**:   - Partnerships existed between PH, NPs, and other community agencies, including non-profit organizations, day cares, elementary schools, university, pediatricians | **Primary Care (PC):**   - PC clinic, PC nurses, PC physician   **Public Health (PH):**   - PH units, PH nurses, PH physician, addictions worker, mental health, social worker, speech/audiology   **Other Partners**:   - A coalition of community organizations, including Community stakeholders and partners, NGOs, outreach services, HIV-AIDS organization, and home care (LPNs) | **Primary care**   - RNs, family physicians, administrators, managers   **Public Health**   - PHNs, managers, PH physicians   **Other Partners**   - occupational therapists, community organizations such as homeless shelters, needle exchange service, organizations working with sex workers |
| **Health Issues Addressed** | - Child health promotion/development for those who do not necessarily have a regular source of care - Social determinants of health (SDOH) - Women’s health | - Homeless or street-involved population and sex trade workers who do not necessarily have a regular source of care - Social determinants of health - Flu prevention, prenatal, STI/HIV screening and management, mental health | - Immunizations for street-involved population |
| **Precipitators of collaboration** | - Saw ways to use resources differently to solve problems in community (NPs provide PC services in community) - Hospital to ensure children with special health care needs in poverty seen by specialists, outreach by hospital | - Saw ways to: reduce emergency room demands; increase access for “hard to reach” - Saw working together would have greater potential for improvements - Outbreak provided opportunity for collaboration | - Proposal for funding accepted. - PC and PH had common goal of improving health care services to this population |
| **Problem being addressed by collaboration** | - Barriers to accessing care -PC services - Helping clients navigate healthcare system (e.g., parents with children with developmental challenges) | - Lack of mental health/PC services - Few MDs willing to take on new patients | - Challenge reaching street-involved population for vaccinations and communicable disease management |
| **Goals** | - Creating resources to treat children and address SDOH - Reducing barriers to accessing PC for vulnerable populations - Establishing relationships with patients - Improving accountabilities - Enhance evidence-based research | - Meeting mental health, addictions, harm reduction needs - Improve access to care for underserviced populations - Provide one-stop shop for care - Establish levels of accountability and performance measurement - Prevent communicable disease spread | - Informal goals are to improve access to care for street-involved population |
| **Key factors influencing success of the collaboration** | **INTRAPERSONAL**  **PERSONAL QUALITIES, KNOWLEDGE AND SKILLS**   - Willing to make personal investment to build relationships - Each person brought unique skill set   **PERSONAL VALUES , BELIEFS AND ATTITUDES**   - Staff made personal sacrifices - Understood building community relationships/trust crucial takes work   **INTERPERSONAL**  **ROLE CLARITY**   - NP job description publicly shared   **EFFECTIVE COMMUNICATION**   - People knew right person to contact - Schedules differed; sometimes difficult to communicate efficiently   **TRUSTING AND INCLUSIVE RELATIONSHIPS**   - Fostered partners/clients relationships by engaging local level - At “community table” regularly   **SHARED VALUES, BELIEFS, AND ATTITUDES**   - Required dedicated individual commitment, belief in collaboration   **ORGANIZATIONAL**  **Clear Mandates, Vision and Goals for collaboration**   - Organizations had differing philosophies; Collaboration could increase with clear vision by PH and PC employers - Difficulty communicating roles to all - Formal partnership   **strategic Coordination and**  **Communication Mechanisms between partners**   - Communication processes differed - Lacked shared filing system - Weekly meetings, but PH not aware   **formal organizational Leaders as collaboration Champions**   - Leadership weak and no buy in; lacked synergy due to busyness and large portfolios - Leadership acting as partners, not bosses; no identifiable leader in charge of partnership - Collaborative leadership style: people took varied leadership roles   **COLLABORATIVE ORGANIZATIONAL CULTURE**   - Ability to adapt to changing demands - Dealt with conflicts case by case   **OPTIMAL USE OF RESOURCES**   - Sharing space in different community organizations/locations - Funding from variety of sources   **OPTIMAL USE OF HUMAN RESOURCES**   - Funding required for backfill positions - Funding for NP role provided   **COLLABORATIVE APPROACHES TO Programs and Services Delivery**   - Difficult to work across organizations - Lacked support for NP role - Client-centred approach - Multidisciplinary partners   **SYSTEMIC**  **GOVERNMENTAL AND REGULATORY POLICIES AND MANDATES FOR COLLABORATION**   - Government did not address root of problem - No collaboration representation at government policy table   **FUNDING MODELS AND FINANCIAL INCENTIVES SUPPORTING COLLABORATION**   - Insufficient systems funding hampered collaborative efforts   **HEALTH SERVICE STRUCTURES THAT PROMOTE COLLABORATION**   - PH not addressing social determinants of health - Worked in silos | **INTRAPERSONAL**  **PERSONAL QUALITIES, KNOWLEDGE AND SKILLS**   - Front line staff capable; team leaders able to provide answers   **PERSONAL VALUES , BELIEFS AND ATTITUDES**   - Collaboration valued; willingness to collaborate to meet common goal(s)   **INTERPERSONAL**  **ROLE CLARITY**   - Unclear delineation of roles - PH and PC silos deter role clarity - Changing PHN roles creates confusion and uncertainty   **EFFECTIVE COMMUNICATION**   - Phone outreach for patient inquiries - Partner communication connected to effectiveness of collaboration - People knew right person to contact   **TRUSTING AND INCLUSIVE RELATIONSHIPS**   - PC and PH positive working relationships; trust, respect, and openness between partners   **SHARED VALUES, BELIEFS, AND ATTITUDES**   - By working closely together, shared values and goals grew organically   **ORGANIZATIONAL**  **Clear Mandates, Vision and Goals for collaboration**   - Uncertainty about/lack of shared goals and vision - PHNs struggled to determine roles   **strategic Coordination and**  **Communication Mechanisms between partners**   - No formal communication processes between PH & PC - Lack of seamless EMR system - Regularly scheduled meetings   **formal organizational Leaders as collaboration Champions**   - Weak presence of leadership - Challenge to know who leaders were; reporting structure unclear - People took informal leader roles   **COLLABORATIVE ORGANIZATIONAL CULTURE**   - PHNs unhappy with changing roles - Collaboration not yet ingrained; some feeling protective of turf - Lack of understanding of the other; valuing work of the other   **OPTIMAL USE OF RESOURCES**   - Lack of flexibility of funding; funding from various sources - Having physical space helpful   **COLLABORATIVE APPROACHES TO Programs and Services Delivery**   - Should have engaged community focused on community development - PH & PC sectors working in silos - Client-centered approach - Multidisciplinary teams brought different individual strengths - Powerful synergy exploring different views, individual and population health perspectives   **SYSTEMIC**  **GOVERNMENTAL AND REGULATORY POLICIES AND MANDATES FOR COLLABORATION**   - Unclear mandates for collaboration   **HARMONIZED INFORMATION AND COMMUNICATION INFRASTRUCTURE**   - No common PC PH standard communication infrastructure   **FUNDING MODELS AND FINANCIAL INCENTIVES SUPPORTING COLLABORATION**   - Competition for funding results in division of sectors   **HEALTH SERVICE STRUCTURES THAT PROMOTE COLLABORATION**   - Provincial changes occurring particularly in PC; perceived to have negative impact on players | **INTRAPERSONAL**  **PERSONAL QUALITIES, KNOWLEDGE AND SKILLS**   - Those involved have confidence and passion for working with marginalized populations   **INTERPERSONAL**  **EFFECTIVE COMMUNICATION**   - Communication is informal; can break down when service delivery needs are high   **TRUSTING AND INCLUSIVE RELATIONSHIPS**   - Former working relationships between PC & PH partners enabled collaboration   **SHARED VALUES, BELIEFS, AND ATTITUDES**   - Partners share passion for equity and social justice   **ORGANIZATIONAL**  **Clear Mandates, Vision and Goals for collaboration**   - No formal agreements; this enabled flexibility and at the same time expansion of the partnership   **strategic Coordination and**  **Communication Mechanisms between partners**   - No formal communication structures in place - Communication occurs on as needed basis   **formal organizational Leaders as collaboration Champions**   - PC and PH administrators support PC and PH philosophies   **OPTIMAL USE OF RESOURCES**   - In-kind resources for collaboration   **SYSTEMIC**  **GOVERNMENTAL AND REGULATORY POLICIES AND MANDATES FOR COLLABORATION**   - Little support from provincial government to date - With support effectiveness of collaboration could be improved |
| **Community Involvement in collaboration** | - Community members are partners in collaboration - Community partners were involved at all stages of collaboration | - Community identified the need for collaboration - Community partners voiced the needs of the community | - Community organizations involved with service provision to the population but not PC–PH collaboration specifically |
| **Key impacts and Outcomes** | - Developed or increased knowledge skills of staff - Improved the information sharing system - Improved patients’ accessibility to care | - Improved accessibility to care - Provided one-stop shopping for patients and provided continuity of care - Improved work processes among staff - Improved relationships | - Improved accessibility to immunizations for hard to reach - Improved data collection regarding immunization in this population - PH provided some training for PC nurses to vaccinate their clients - Improved responsiveness to epidemics e.g. H1N1 |
| **Research and/ or Evaluation** | - Research was conducted on collaboration and was integral - No evidence of evaluation being done by management - Various evaluations by partners (e.g. process evaluation, satisfaction surveys, and evaluation of collaboration’s model of access) conducted - Interdisciplinary research team - Research is central part of partnership; findings presented at conferences; published papers exist | - Little being done in evaluation and/or research; objectives identified to assess success of project | - No formal evaluation of PH-PC collaboration |

# 
